# Chlorido{*N* ^2^,*N* ^6^-dibenzyl-*N* ^2^,*N* ^6^-bis­[(diphenyl­phosphan­yl)meth­yl]pyridine-2,6-diamine}­methyl­platinum(II)

**DOI:** 10.1107/S1600536810038134

**Published:** 2010-09-30

**Authors:** Zi-Jia Wang, Xiao-Xi Wang, Chong-Qing Wan

**Affiliations:** a Department of Chemistry, Capital Normal University, Beijing 100048, People’s Republic of China

## Abstract

In the title mononuclear complex, [Pt(CH_3_)Cl(C_45_H_41_N_3_P_2_)], the pyridine-2,6-diamine ligand can be viewed as a centrosymmetric motif having two pendant *N*-benzyl-*N*-[(diphenyl­phosphan­yl)meth­yl] arms, the two P atoms of which chelate to the Pt^II^ ion, forming a ten-membered metallocycle. A distorted square-planar coordination geometry around the Pt^II^ atom is completed by a methyl ligand and a chloride ion. The packing between the mononuclear units is achieved through C—H⋯π inter­actions, which link the mol­ecules into chains along the *c* axis.

## Related literature

For coordination complexes with hemilabile tridentate ligands with P*X*P (*X* = C, N, O, S, and As) donor sets, see: Ainscough *et al.* (2004 ▶); Song *et al.* (2002 ▶); Kunz *et al.* (2010 ▶); Wang *et al.* (2010 ▶); Zhang & Cheng (1996 ▶). For a coordination complex of the 2,6-bis­(*N*-benzyl-*N*-diphenyl­phosphinomethyl­amino)­pyri­dine ligand, see: Li *et al.* (2005 ▶). For C—H(benzene)⋯π inter­actions, see: Umezawa *et al.* (1998 ▶). 
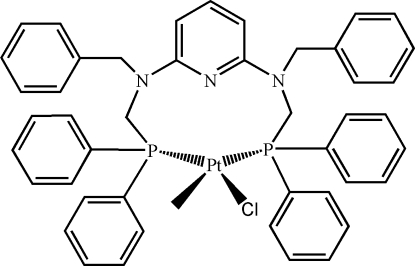


## Experimental

### 

#### Crystal data

[Pt(CH_3_)Cl(C_45_H_41_N_3_P_2_)]
*M* *_r_* = 931.32
Orthorhombic, 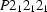

*a* = 15.3515 (9) Å
*b* = 15.9294 (10) Å
*c* = 16.5197 (10) Å
*V* = 4039.7 (4) Å^3^
*Z* = 4
Mo *K*α radiation
μ = 3.66 mm^−1^
*T* = 293 K
0.32 × 0.24 × 0.16 mm

#### Data collection

Bruker APEXII CCD area-detector diffractometer
Absorption correction: multi-scan (*SADABS*; Bruker, 2007 ▶) *T*_min_ = 0.640, *T*_max_ = 1.000
21886 measured reflections
7108 independent reflections
6498 reflections with *I* > 2σ(*I*)
*R*_int_ = 0.029

#### Refinement

*R*[*F* ^2^ > 2σ(*F* ^2^)] = 0.024
*wR*(*F* ^2^) = 0.050
*S* = 0.97
7108 reflections
479 parameters
H-atom parameters constrained
Δρ_max_ = 0.74 e Å^−3^
Δρ_min_ = −0.38 e Å^−3^
Absolute structure: Flack (1983 ▶), 3125 Friedel pairs
Flack parameter: −0.010 (4)

### 

Data collection: *APEX2* (Bruker, 2007 ▶); cell refinement: *APEX2* and *SAINT* (Bruker, 2007 ▶); data reduction: *SAINT*; program(s) used to solve structure: *SHELXS97* (Sheldrick, 2008 ▶); program(s) used to refine structure: *SHELXL97* (Sheldrick, 2008 ▶); molecular graphics: *SHELXTL* (Sheldrick, 2008 ▶); software used to prepare material for publication: *SHELXTL* and *PLATON* (Spek, 2009 ▶).

## Supplementary Material

Crystal structure: contains datablocks I, global. DOI: 10.1107/S1600536810038134/bq2234sup1.cif

Structure factors: contains datablocks I. DOI: 10.1107/S1600536810038134/bq2234Isup2.hkl

Additional supplementary materials:  crystallographic information; 3D view; checkCIF report

## Figures and Tables

**Table 1:** Selected bond lengths (Å)

Pt1—C46	2.172 (3)
Pt1—P1	2.2105 (11)
Pt1—P2	2.3153 (10)
Pt1—Cl1	2.3663 (12)

**Table 2:** Hydrogen-bond geometry (Å, °) *Cg*1 is the centroid of the C15–C20 benzene ring.

*D*—H⋯*A*	*D*—H	H⋯*A*	*D*⋯*A*	*D*—H⋯*A*
C5—H5⋯*Cg*1^i^	0.93	3.00	3.812 (2)	146
C26—H26*B*⋯*Cg*1^ii^	0.97	2.96	3.807 (2)	147

Symmetry codes: (i) 
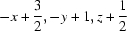
; (ii) 
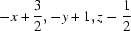
.

**Fig. 1.**
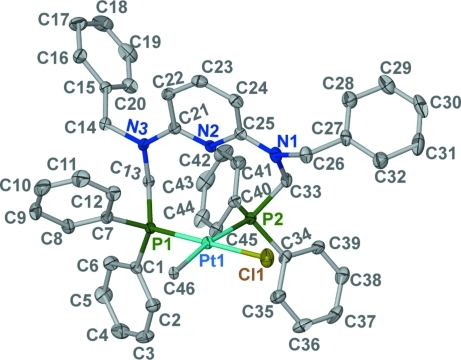
The atom-numbering scheme of the title complex I.Displacement ellipsoids are drawn at the 30% probability level and H atoms are omitted for clarity.

**Fig. 2.**
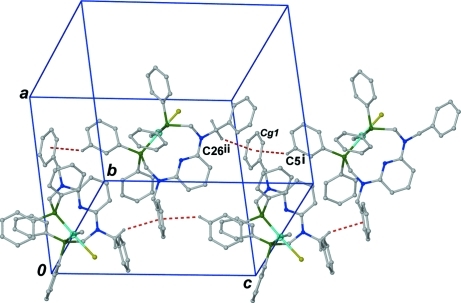
The C—H(benzene)···π interactions between the mononuclear Pt(L)MeCl units arranged along the c direction. The red-dashed lines indicate the C—H···π interactions, while the Cg1 presents ring of C15-C20 (benzene). symmetry codes: i -x + 1.5, -y + 1, z + 0.5; ii -x + 1.5, -y + 1, z - 0.5

## supplementary crystallographic information

### Comment

The hemilabile tridentate ligands with PXP (X = C, N, O, S, and As) donor sets have attracted particular attention for their coordinative extensibility and structural diversity with transition metals (Ainscough *et al.*, 2004; Song, *et al.*, 2002; Kunz *et al.*, 2010; Wang, *et al.*, 2010), as well as the catalytic property of their metal complexes (Zhang *et al.*, 1996).

In the present context, we report a new complex of 2,6-bis(N-benzyl-N-diphenylphosphinomethylamino)pyridine ligand (abbreviated as L), namely Pt(L)MeCl, (I). In the title complex, two phosphine atoms of the pendant diphenylphosinomethylamino arms bond to the square-planar coordinated Pt^II^ ion at the *cis* sites, generating a 10-membered ring similar to that of the reported Pt(L)Cl_2_.CH_2_Cl_2_ (Li *et al.*, 2005), as shown in Fig. 1. The mononuclear units are arranged along the c direction and interconnect through C-H(methylene)..π and C—H(benzene)···π interactions (Umezawa *et al.*, 1998) to form a chain. As shown in Fig. 2, the C5^i^—H^i^···Cg1 contact exhibits a C5^i^···centroid distance of 3.812 (2) Å and a C5^i^—H^i^···centroid angle of 146°, while the C26^ii^—H(methylene) interaction has a C26^ii^···centroid separation of 3.807 (2) Å and a C26^ii^—H^ii^···centroid angle of 147° [Cg1 presents ring of C15-C20 (benzene), symmetry codes: i -*x* + 1.5, -*y* + 1, *z* + 0.5; ii -*x* + 1.5, -*y* + 1, *z* - 0.5].

### Experimental

The 2,6-bis(N-benzyl-N-diphenylphosphinomethylamino)pyridine (L) was synthesized through the procedure as the literature (Li *et al.*, 2005). The solid of Pt(COD)MeCl (0.210 g, 0.60 mmol) was added to the solution of L (0.411 g, 0.6 mmol) in CH_2_Cl_2_ (30 ml) and the mixture was stirred for one hour at room temperature. The yellow solvent was removed under vacuum and the title complex was obtained as a yellow powder, the block like crystals of which were obtained after four days by recrystallization from CH_2_Cl_2_/n-hexane (0.447 g, 80% yield).

### Refinement

The hydrogen atoms were placed in idealized positions and allowed to ride on the relevant carbon atoms, with C—H = 0.93, 0.97 and 0.96 Å for aryl, methylene and methyl hydrogens, respectively. *U_iso_*(H) = 1.2*U_eq_*(C).

### Crystal data

**Table:**

[Pt(CH_3_)Cl(C_45_H_41_N_3_P_2_)]	*Z* = 4
*M**_r_* = 931.32	*F*(000) = 1864
Orthorhombic, *P*2_1_2_1_2_1_	*D*_x_ = 1.531 Mg m^−^^3^
Hall symbol: P 2ac 2ab	Mo *K*α radiation, λ = 0.71073 Å
*a* = 15.3515 (9) Å	µ = 3.66 mm^−^^1^
*b* = 15.9294 (10) Å	*T* = 293 K
*c* = 16.5197 (10) Å	Block, colorless
*V* = 4039.7 (4) Å^3^	0.32 × 0.24 × 0.16 mm

### Data collection

**Table:**

Bruker APEXII CCD area-detector diffractometer	7108 independent reflections
Radiation source: fine-focus sealed tube	6498 reflections with *I* > 2σ(*I*)
graphite	*R*_int_ = 0.029
ω scans	θ_max_ = 25.0°, θ_min_ = 1.8°
Absorption correction: multi-scan (*SADABS*; Bruker, 2007)	*h* = −15→18
*T*_min_ = 0.640, *T*_max_ = 1.000	*k* = −18→18
21886 measured reflections	*l* = −17→19

### Refinement

**Table:**

Refinement on *F*^2^	Secondary atom site location: difference Fourier map
Least-squares matrix: full	Hydrogen site location: inferred from neighbouring sites
*R*[*F*^2^ > 2σ(*F*^2^)] = 0.024	H-atom parameters constrained
*wR*(*F*^2^) = 0.050	*w* = 1/[σ^2^(*F*_o_^2^) + (0.*P*)^2^] *P* = (*F*_o_^2^ + 2*F*_c_^2^)/3
*S* = 0.97	(Δ/σ)_max_ = 0.003
7108 reflections	Δρ_max_ = 0.74 e Å^−^^3^
479 parameters	Δρ_min_ = −0.38 e Å^−^^3^
0 restraints	Absolute structure: Flack (1983), 3125 Friedel pairs
Primary atom site location: structure-invariant direct methods	Flack parameter: −0.010 (4)

### Special details

**Table:**

Geometry. All esds (except the esd in the dihedral angle between two l.s. planes) are estimated using the full covariance matrix. The cell esds are taken into account individually in the estimation of esds in distances, angles and torsion angles; correlations between esds in cell parameters are only used when they are defined by crystal symmetry. An approximate (isotropic) treatment of cell esds is used for estimating esds involving l.s. planes.
Refinement. Refinement of F^2^ against ALL reflections. The weighted R-factor wR and goodness of fit S are based on F^2^, conventional R-factors R are based on F, with F set to zero for negative F^2^. The threshold expression of F^2^ > 2sigma(F^2^) is used only for calculating R-factors(gt) etc. and is not relevant to the choice of reflections for refinement. R-factors based on F^2^ are statistically about twice as large as those based on F, and R- factors based on ALL data will be even larger.

### Fractional atomic coordinates and isotropic or equivalent isotropic displacement parameters (Å^2^)

**Table:**

	*x*	*y*	*z*	*U*_iso_*/*U*_eq_	
Pt1	0.442088 (10)	0.536151 (10)	0.964197 (10)	0.03462 (5)	
Cl1	0.33715 (9)	0.50166 (8)	1.06371 (8)	0.0633 (4)	
P1	0.54103 (7)	0.57645 (7)	0.87509 (7)	0.0328 (3)	
P2	0.47428 (6)	0.39415 (6)	0.96383 (8)	0.0344 (2)	
N1	0.5395 (2)	0.4046 (2)	1.1248 (2)	0.0444 (9)	
N2	0.62990 (18)	0.47138 (19)	1.03402 (19)	0.0340 (7)	
N3	0.7081 (2)	0.5456 (2)	0.93740 (19)	0.0386 (9)	
C1	0.4996 (3)	0.5651 (2)	0.7722 (3)	0.0371 (10)	
C2	0.4096 (3)	0.5615 (3)	0.7627 (3)	0.0513 (13)	
H2	0.3739	0.5667	0.8079	0.062*	
C3	0.3728 (3)	0.5505 (4)	0.6874 (3)	0.0627 (15)	
H3	0.3125	0.5483	0.6821	0.075*	
C4	0.4240 (4)	0.5428 (4)	0.6211 (3)	0.0686 (14)	
H4	0.3990	0.5348	0.5704	0.082*	
C5	0.5120 (4)	0.5466 (4)	0.6290 (3)	0.0693 (15)	
H5	0.5469	0.5418	0.5832	0.083*	
C6	0.5504 (3)	0.5576 (3)	0.7034 (3)	0.0568 (13)	
H6	0.6108	0.5600	0.7075	0.068*	
C7	0.5844 (3)	0.6819 (2)	0.8848 (3)	0.0397 (10)	
C8	0.6100 (3)	0.7296 (3)	0.8197 (3)	0.0525 (12)	
H8	0.5969	0.7123	0.7674	0.063*	
C9	0.6558 (4)	0.8044 (3)	0.8325 (4)	0.0717 (17)	
H9	0.6739	0.8365	0.7886	0.086*	
C10	0.6739 (4)	0.8304 (3)	0.9096 (5)	0.0745 (18)	
H10	0.7040	0.8803	0.9180	0.089*	
C11	0.6482 (3)	0.7836 (3)	0.9737 (4)	0.0709 (16)	
H11	0.6613	0.8015	1.0259	0.085*	
C12	0.6028 (3)	0.7101 (3)	0.9627 (3)	0.0506 (11)	
H12	0.5844	0.6791	1.0072	0.061*	
C13	0.6476 (2)	0.5207 (3)	0.8754 (2)	0.0387 (10)	
H13A	0.6366	0.4610	0.8811	0.046*	
H13B	0.6751	0.5291	0.8231	0.046*	
C14	0.7841 (3)	0.5946 (3)	0.9142 (3)	0.0430 (11)	
H14A	0.7908	0.6408	0.9519	0.052*	
H14B	0.7742	0.6184	0.8609	0.052*	
C15	0.8671 (3)	0.5450 (3)	0.9125 (2)	0.0427 (10)	
C16	0.9462 (3)	0.5834 (3)	0.9267 (3)	0.0582 (12)	
H16	0.9473	0.6399	0.9408	0.070*	
C17	1.0234 (3)	0.5404 (5)	0.9205 (3)	0.0762 (16)	
H17	1.0761	0.5674	0.9303	0.091*	
C18	1.0216 (4)	0.4565 (6)	0.8994 (4)	0.087 (2)	
H18	1.0736	0.4269	0.8943	0.104*	
C19	0.9465 (5)	0.4176 (4)	0.8865 (3)	0.0764 (17)	
H19	0.9461	0.3608	0.8735	0.092*	
C20	0.8677 (3)	0.4615 (4)	0.8924 (3)	0.0580 (12)	
H20	0.8154	0.4337	0.8827	0.070*	
C21	0.6965 (2)	0.5226 (2)	1.0174 (2)	0.0369 (10)	
C22	0.7514 (3)	0.5538 (3)	1.0782 (3)	0.0425 (11)	
H22	0.7995	0.5868	1.0656	0.051*	
C23	0.7319 (3)	0.5341 (3)	1.1561 (3)	0.0480 (11)	
H23	0.7677	0.5538	1.1974	0.058*	
C24	0.6610 (3)	0.4863 (3)	1.1754 (3)	0.0462 (12)	
H24	0.6466	0.4752	1.2290	0.055*	
C25	0.6110 (3)	0.4545 (3)	1.1117 (2)	0.0370 (9)	
C26	0.4923 (3)	0.4046 (3)	1.2028 (3)	0.0496 (12)	
H26A	0.4324	0.4212	1.1933	0.060*	
H26B	0.5185	0.4461	1.2382	0.060*	
C27	0.4930 (3)	0.3215 (3)	1.2446 (3)	0.0458 (12)	
C28	0.5690 (4)	0.2808 (3)	1.2634 (3)	0.0626 (14)	
H28	0.6221	0.3046	1.2489	0.075*	
C29	0.5676 (5)	0.2044 (4)	1.3036 (3)	0.0746 (16)	
H29	0.6199	0.1774	1.3151	0.089*	
C30	0.4926 (6)	0.1689 (4)	1.3263 (4)	0.086 (2)	
H30	0.4927	0.1178	1.3535	0.104*	
C31	0.4153 (5)	0.2087 (4)	1.3089 (4)	0.090 (2)	
H31	0.3628	0.1847	1.3249	0.108*	
C32	0.4153 (4)	0.2852 (3)	1.2672 (3)	0.0660 (15)	
H32	0.3629	0.3115	1.2548	0.079*	
C33	0.5016 (3)	0.3507 (3)	1.0645 (3)	0.0434 (11)	
H33A	0.4486	0.3274	1.0872	0.052*	
H33B	0.5414	0.3042	1.0561	0.052*	
C34	0.3790 (3)	0.3301 (3)	0.9376 (3)	0.0412 (11)	
C35	0.3074 (3)	0.3654 (3)	0.9016 (3)	0.0549 (13)	
H35	0.3056	0.4232	0.8936	0.066*	
C36	0.2383 (3)	0.3169 (4)	0.8772 (4)	0.0724 (16)	
H36	0.1905	0.3414	0.8521	0.087*	
C37	0.2406 (4)	0.2315 (4)	0.8903 (4)	0.0698 (16)	
H37	0.1936	0.1984	0.8747	0.084*	
C38	0.3104 (4)	0.1956 (3)	0.9257 (3)	0.0635 (15)	
H38	0.3116	0.1379	0.9344	0.076*	
C39	0.3789 (3)	0.2437 (3)	0.9485 (3)	0.0508 (12)	
H39	0.4271	0.2182	0.9722	0.061*	
C40	0.5494 (3)	0.3437 (2)	0.8929 (3)	0.0371 (10)	
C41	0.5277 (3)	0.3486 (3)	0.8112 (3)	0.0501 (12)	
H41	0.4761	0.3743	0.7952	0.060*	
C42	0.5840 (4)	0.3148 (3)	0.7537 (3)	0.0637 (15)	
H42	0.5696	0.3183	0.6991	0.076*	
C43	0.6587 (4)	0.2773 (4)	0.7757 (5)	0.0791 (19)	
H43	0.6955	0.2553	0.7364	0.095*	
C44	0.6806 (4)	0.2714 (3)	0.8551 (5)	0.0746 (18)	
H44	0.7323	0.2450	0.8700	0.089*	
C45	0.6265 (3)	0.3044 (3)	0.9140 (3)	0.0541 (13)	
H45	0.6420	0.3002	0.9683	0.065*	
C46	0.3910 (2)	0.66327 (19)	0.9668 (3)	0.0264 (8)	
H46A	0.4009	0.6874	1.0193	0.040*	
H46B	0.3296	0.6621	0.9558	0.040*	
H46C	0.4199	0.6965	0.9264	0.040*	

### Atomic displacement parameters (Å^2^)

**Table:**

	*U*^11^	*U*^22^	*U*^33^	*U*^12^	*U*^13^	*U*^23^
Pt1	0.03517 (8)	0.03335 (7)	0.03532 (8)	0.00224 (8)	0.00569 (8)	0.00014 (9)
Cl1	0.0630 (8)	0.0570 (6)	0.0698 (9)	0.0094 (6)	0.0314 (7)	0.0093 (6)
P1	0.0343 (7)	0.0330 (5)	0.0310 (6)	−0.0013 (4)	0.0000 (5)	0.0017 (5)
P2	0.0359 (5)	0.0318 (5)	0.0354 (6)	−0.0028 (4)	0.0006 (6)	−0.0013 (6)
N1	0.063 (3)	0.044 (2)	0.0255 (19)	−0.0086 (18)	0.0047 (17)	−0.0020 (16)
N2	0.0392 (17)	0.0321 (16)	0.0306 (17)	−0.0007 (15)	−0.0022 (16)	0.002 (2)
N3	0.0324 (18)	0.046 (2)	0.037 (2)	−0.0084 (17)	0.0007 (14)	0.0068 (18)
C1	0.048 (3)	0.033 (2)	0.031 (2)	−0.0003 (19)	−0.005 (2)	0.0005 (18)
C2	0.047 (3)	0.058 (3)	0.049 (3)	−0.001 (2)	−0.001 (2)	−0.001 (2)
C3	0.048 (3)	0.080 (4)	0.060 (3)	−0.005 (3)	−0.016 (3)	−0.011 (3)
C4	0.078 (4)	0.084 (4)	0.044 (3)	−0.002 (3)	−0.016 (3)	−0.007 (3)
C5	0.080 (4)	0.095 (4)	0.033 (3)	−0.006 (4)	0.002 (3)	−0.008 (3)
C6	0.053 (3)	0.080 (4)	0.037 (3)	−0.003 (3)	0.001 (3)	0.002 (2)
C7	0.038 (3)	0.033 (2)	0.047 (3)	−0.0015 (18)	0.001 (2)	−0.001 (2)
C8	0.057 (3)	0.046 (3)	0.054 (3)	−0.006 (2)	−0.004 (3)	0.008 (2)
C9	0.066 (4)	0.047 (3)	0.102 (5)	−0.012 (3)	0.002 (4)	0.027 (3)
C10	0.063 (4)	0.044 (3)	0.117 (6)	−0.016 (3)	−0.016 (4)	−0.006 (3)
C11	0.076 (4)	0.051 (3)	0.085 (4)	−0.005 (3)	−0.016 (4)	−0.017 (3)
C12	0.054 (3)	0.042 (2)	0.055 (3)	−0.002 (2)	−0.002 (3)	−0.007 (3)
C13	0.040 (2)	0.041 (3)	0.036 (2)	−0.0030 (19)	0.0029 (19)	0.005 (2)
C14	0.039 (2)	0.044 (3)	0.046 (3)	−0.005 (2)	0.000 (2)	0.007 (2)
C15	0.041 (2)	0.056 (3)	0.032 (2)	−0.003 (2)	0.0002 (18)	0.009 (2)
C16	0.048 (3)	0.073 (3)	0.053 (3)	−0.013 (3)	−0.001 (3)	0.011 (2)
C17	0.042 (3)	0.115 (5)	0.072 (4)	0.007 (4)	0.001 (3)	0.018 (4)
C18	0.061 (4)	0.135 (7)	0.065 (4)	0.044 (5)	0.016 (3)	0.018 (5)
C19	0.096 (5)	0.080 (4)	0.054 (3)	0.035 (4)	0.016 (4)	0.004 (3)
C20	0.065 (3)	0.060 (3)	0.049 (3)	0.006 (3)	−0.004 (2)	0.002 (3)
C21	0.038 (2)	0.029 (2)	0.044 (3)	0.0050 (18)	−0.0004 (18)	−0.0015 (19)
C22	0.040 (2)	0.041 (3)	0.046 (3)	−0.0045 (19)	−0.008 (2)	−0.004 (2)
C23	0.051 (3)	0.046 (2)	0.048 (3)	0.005 (3)	−0.015 (2)	−0.008 (3)
C24	0.062 (3)	0.045 (3)	0.032 (2)	0.004 (2)	−0.007 (2)	−0.003 (2)
C25	0.045 (2)	0.030 (2)	0.035 (2)	0.003 (2)	−0.0004 (19)	−0.001 (2)
C26	0.062 (3)	0.051 (3)	0.036 (3)	−0.002 (2)	0.013 (2)	0.001 (2)
C27	0.062 (3)	0.048 (3)	0.027 (2)	−0.012 (2)	0.008 (2)	0.002 (2)
C28	0.069 (4)	0.065 (3)	0.054 (3)	0.000 (3)	0.008 (3)	0.005 (3)
C29	0.104 (5)	0.070 (4)	0.049 (3)	0.013 (4)	0.003 (4)	0.011 (3)
C30	0.148 (7)	0.061 (4)	0.051 (4)	−0.014 (4)	0.000 (4)	0.019 (3)
C31	0.107 (6)	0.079 (4)	0.084 (5)	−0.041 (4)	0.015 (4)	0.018 (4)
C32	0.070 (4)	0.073 (4)	0.054 (3)	−0.014 (3)	−0.002 (3)	0.007 (3)
C33	0.053 (3)	0.037 (2)	0.040 (3)	−0.012 (2)	−0.001 (2)	0.004 (2)
C34	0.034 (2)	0.048 (3)	0.042 (3)	−0.004 (2)	0.008 (2)	−0.006 (2)
C35	0.043 (3)	0.050 (3)	0.071 (4)	−0.003 (2)	−0.006 (3)	−0.006 (3)
C36	0.039 (3)	0.085 (4)	0.094 (5)	−0.004 (3)	−0.008 (3)	−0.014 (4)
C37	0.055 (4)	0.078 (4)	0.077 (4)	−0.034 (3)	0.008 (3)	−0.020 (3)
C38	0.070 (4)	0.052 (3)	0.068 (4)	−0.022 (3)	0.002 (3)	−0.008 (3)
C39	0.046 (3)	0.041 (2)	0.066 (4)	−0.009 (2)	−0.009 (2)	0.001 (2)
C40	0.034 (2)	0.034 (2)	0.043 (3)	−0.001 (2)	0.000 (2)	−0.0075 (19)
C41	0.048 (3)	0.050 (3)	0.052 (3)	−0.014 (2)	0.008 (2)	−0.010 (2)
C42	0.066 (4)	0.070 (3)	0.055 (3)	−0.020 (3)	0.016 (3)	−0.022 (3)
C43	0.066 (4)	0.071 (4)	0.100 (5)	−0.009 (3)	0.030 (4)	−0.039 (4)
C44	0.050 (3)	0.061 (4)	0.112 (6)	0.013 (3)	0.012 (4)	−0.019 (4)
C45	0.048 (3)	0.049 (3)	0.065 (3)	0.003 (2)	−0.001 (3)	−0.004 (3)
C46	0.0311 (19)	0.0184 (16)	0.030 (2)	0.0069 (14)	0.011 (2)	−0.0014 (18)

### Geometric parameters (Å, °)

**Table:**

Pt1—C46	2.172 (3)	C18—H18	0.9300
Pt1—P1	2.2105 (11)	C19—C20	1.400 (7)
Pt1—P2	2.3153 (10)	C19—H19	0.9300
Pt1—Cl1	2.3663 (12)	C20—H20	0.9300
P1—C7	1.814 (4)	C21—C22	1.402 (6)
P1—C1	1.824 (4)	C22—C23	1.358 (6)
P1—C13	1.862 (4)	C22—H22	0.9300
P2—C40	1.830 (4)	C23—C24	1.366 (6)
P2—C34	1.836 (4)	C23—H23	0.9300
P2—C33	1.850 (4)	C24—C25	1.397 (6)
N1—C25	1.373 (5)	C24—H24	0.9300
N1—C33	1.438 (5)	C26—C27	1.493 (6)
N1—C26	1.478 (5)	C26—H26A	0.9700
N2—C21	1.336 (5)	C26—H26B	0.9700
N2—C25	1.343 (5)	C27—C28	1.370 (7)
N3—C21	1.383 (5)	C27—C32	1.377 (7)
N3—C13	1.439 (5)	C28—C29	1.386 (7)
N3—C14	1.455 (5)	C28—H28	0.9300
C1—C6	1.384 (6)	C29—C30	1.336 (9)
C1—C2	1.390 (6)	C29—H29	0.9300
C2—C3	1.378 (6)	C30—C31	1.377 (9)
C2—H2	0.9300	C30—H30	0.9300
C3—C4	1.354 (7)	C31—C32	1.400 (8)
C3—H3	0.9300	C31—H31	0.9300
C4—C5	1.359 (7)	C32—H32	0.9300
C4—H4	0.9300	C33—H33A	0.9700
C5—C6	1.374 (6)	C33—H33B	0.9700
C5—H5	0.9300	C34—C35	1.370 (6)
C6—H6	0.9300	C34—C39	1.387 (6)
C7—C8	1.375 (6)	C35—C36	1.373 (7)
C7—C12	1.390 (7)	C35—H35	0.9300
C8—C9	1.400 (7)	C36—C37	1.377 (8)
C8—H8	0.9300	C36—H36	0.9300
C9—C10	1.368 (8)	C37—C38	1.349 (8)
C9—H9	0.9300	C37—H37	0.9300
C10—C11	1.353 (8)	C38—C39	1.354 (6)
C10—H10	0.9300	C38—H38	0.9300
C11—C12	1.376 (6)	C39—H39	0.9300
C11—H11	0.9300	C40—C45	1.384 (6)
C12—H12	0.9300	C40—C41	1.392 (6)
C13—H13A	0.9700	C41—C42	1.393 (7)
C13—H13B	0.9700	C41—H41	0.9300
C14—C15	1.499 (6)	C42—C43	1.343 (8)
C14—H14A	0.9700	C42—H42	0.9300
C14—H14B	0.9700	C43—C44	1.358 (8)
C15—C20	1.371 (7)	C43—H43	0.9300
C15—C16	1.380 (6)	C44—C45	1.384 (7)
C16—C17	1.371 (7)	C44—H44	0.9300
C16—H16	0.9300	C45—H45	0.9300
C17—C18	1.382 (10)	C46—H46A	0.9600
C17—H17	0.9300	C46—H46B	0.9600
C18—C19	1.327 (9)	C46—H46C	0.9600
			
C46—Pt1—P1	89.45 (10)	C15—C20—H20	119.8
C46—Pt1—P2	171.11 (9)	C19—C20—H20	119.8
P1—Pt1—P2	97.77 (4)	N2—C21—N3	117.2 (4)
C46—Pt1—Cl1	87.54 (10)	N2—C21—C22	121.9 (4)
P1—Pt1—Cl1	176.26 (4)	N3—C21—C22	120.8 (4)
P2—Pt1—Cl1	85.43 (4)	C23—C22—C21	117.7 (4)
C7—P1—C1	107.6 (2)	C23—C22—H22	121.2
C7—P1—C13	96.84 (18)	C21—C22—H22	121.2
C1—P1—C13	105.16 (19)	C22—C23—C24	121.7 (4)
C7—P1—Pt1	117.51 (15)	C22—C23—H23	119.1
C1—P1—Pt1	110.60 (15)	C24—C23—H23	119.1
C13—P1—Pt1	117.62 (13)	C23—C24—C25	117.6 (4)
C40—P2—C34	96.12 (19)	C23—C24—H24	121.2
C40—P2—C33	105.6 (2)	C25—C24—H24	121.2
C34—P2—C33	100.7 (2)	N2—C25—N1	116.1 (3)
C40—P2—Pt1	124.42 (14)	N2—C25—C24	121.9 (4)
C34—P2—Pt1	111.95 (14)	N1—C25—C24	122.0 (4)
C33—P2—Pt1	114.30 (15)	N1—C26—C27	113.6 (4)
C25—N1—C33	124.0 (3)	N1—C26—H26A	108.9
C25—N1—C26	122.0 (3)	C27—C26—H26A	108.9
C33—N1—C26	114.0 (4)	N1—C26—H26B	108.9
C21—N2—C25	119.0 (3)	C27—C26—H26B	108.9
C21—N3—C13	121.6 (3)	H26A—C26—H26B	107.7
C21—N3—C14	119.9 (3)	C28—C27—C32	118.6 (5)
C13—N3—C14	118.6 (3)	C28—C27—C26	122.0 (5)
C6—C1—C2	117.7 (4)	C32—C27—C26	119.4 (5)
C6—C1—P1	125.2 (4)	C27—C28—C29	120.7 (6)
C2—C1—P1	117.1 (4)	C27—C28—H28	119.7
C3—C2—C1	120.9 (5)	C29—C28—H28	119.7
C3—C2—H2	119.5	C30—C29—C28	121.3 (7)
C1—C2—H2	119.5	C30—C29—H29	119.3
C4—C3—C2	120.3 (5)	C28—C29—H29	119.3
C4—C3—H3	119.9	C29—C30—C31	119.3 (6)
C2—C3—H3	119.9	C29—C30—H30	120.4
C3—C4—C5	119.7 (5)	C31—C30—H30	120.4
C3—C4—H4	120.2	C30—C31—C32	120.2 (6)
C5—C4—H4	120.2	C30—C31—H31	119.9
C4—C5—C6	121.3 (5)	C32—C31—H31	119.9
C4—C5—H5	119.4	C27—C32—C31	119.9 (6)
C6—C5—H5	119.4	C27—C32—H32	120.0
C5—C6—C1	120.2 (5)	C31—C32—H32	120.0
C5—C6—H6	119.9	N1—C33—P2	119.4 (3)
C1—C6—H6	119.9	N1—C33—H33A	107.5
C8—C7—C12	119.2 (4)	P2—C33—H33A	107.5
C8—C7—P1	123.2 (4)	N1—C33—H33B	107.5
C12—C7—P1	117.1 (3)	P2—C33—H33B	107.5
C7—C8—C9	119.7 (5)	H33A—C33—H33B	107.0
C7—C8—H8	120.1	C35—C34—C39	117.6 (4)
C9—C8—H8	120.1	C35—C34—P2	120.9 (3)
C10—C9—C8	120.0 (6)	C39—C34—P2	121.4 (3)
C10—C9—H9	120.0	C34—C35—C36	121.0 (5)
C8—C9—H9	120.0	C34—C35—H35	119.5
C11—C10—C9	120.2 (5)	C36—C35—H35	119.5
C11—C10—H10	119.9	C35—C36—C37	119.4 (5)
C9—C10—H10	119.9	C35—C36—H36	120.3
C10—C11—C12	120.9 (6)	C37—C36—H36	120.3
C10—C11—H11	119.6	C38—C37—C36	120.5 (5)
C12—C11—H11	119.6	C38—C37—H37	119.8
C11—C12—C7	120.0 (5)	C36—C37—H37	119.8
C11—C12—H12	120.0	C37—C38—C39	119.8 (5)
C7—C12—H12	120.0	C37—C38—H38	120.1
N3—C13—P1	115.9 (3)	C39—C38—H38	120.1
N3—C13—H13A	108.3	C38—C39—C34	121.7 (5)
P1—C13—H13A	108.3	C38—C39—H39	119.1
N3—C13—H13B	108.3	C34—C39—H39	119.1
P1—C13—H13B	108.3	C45—C40—C41	118.3 (4)
H13A—C13—H13B	107.4	C45—C40—P2	125.2 (4)
N3—C14—C15	113.8 (3)	C41—C40—P2	116.5 (3)
N3—C14—H14A	108.8	C40—C41—C42	119.5 (5)
C15—C14—H14A	108.8	C40—C41—H41	120.3
N3—C14—H14B	108.8	C42—C41—H41	120.3
C15—C14—H14B	108.8	C43—C42—C41	121.1 (6)
H14A—C14—H14B	107.7	C43—C42—H42	119.5
C20—C15—C16	117.7 (4)	C41—C42—H42	119.5
C20—C15—C14	121.4 (4)	C42—C43—C44	120.3 (6)
C16—C15—C14	120.7 (5)	C42—C43—H43	119.8
C17—C16—C15	121.8 (5)	C44—C43—H43	119.8
C17—C16—H16	119.1	C43—C44—C45	120.3 (6)
C15—C16—H16	119.1	C43—C44—H44	119.9
C16—C17—C18	119.0 (6)	C45—C44—H44	119.9
C16—C17—H17	120.5	C40—C45—C44	120.5 (5)
C18—C17—H17	120.5	C40—C45—H45	119.7
C19—C18—C17	120.6 (6)	C44—C45—H45	119.7
C19—C18—H18	119.7	Pt1—C46—H46A	109.5
C17—C18—H18	119.7	Pt1—C46—H46B	109.5
C18—C19—C20	120.5 (6)	H46A—C46—H46B	109.5
C18—C19—H19	119.8	Pt1—C46—H46C	109.5
C20—C19—H19	119.8	H46A—C46—H46C	109.5
C15—C20—C19	120.4 (5)	H46B—C46—H46C	109.5
			
C46—Pt1—P1—C7	34.8 (2)	C14—N3—C21—N2	−174.1 (3)
P2—Pt1—P1—C7	−150.40 (16)	C13—N3—C21—C22	−174.0 (4)
C46—Pt1—P1—C1	−89.30 (18)	C14—N3—C21—C22	7.4 (6)
P2—Pt1—P1—C1	85.50 (14)	N2—C21—C22—C23	−3.8 (6)
C46—Pt1—P1—C13	149.89 (18)	N3—C21—C22—C23	174.7 (4)
P2—Pt1—P1—C13	−35.31 (15)	C21—C22—C23—C24	−0.5 (7)
P1—Pt1—P2—C40	−8.50 (19)	C22—C23—C24—C25	3.0 (7)
Cl1—Pt1—P2—C40	173.44 (18)	C21—N2—C25—N1	177.0 (3)
P1—Pt1—P2—C34	−123.05 (15)	C21—N2—C25—C24	−2.6 (6)
Cl1—Pt1—P2—C34	58.88 (15)	C33—N1—C25—N2	19.7 (5)
P1—Pt1—P2—C33	123.31 (17)	C26—N1—C25—N2	−158.0 (4)
Cl1—Pt1—P2—C33	−54.75 (17)	C33—N1—C25—C24	−160.7 (4)
C7—P1—C1—C6	72.9 (4)	C26—N1—C25—C24	21.6 (6)
C13—P1—C1—C6	−29.6 (4)	C23—C24—C25—N2	−1.5 (6)
Pt1—P1—C1—C6	−157.6 (4)	C23—C24—C25—N1	178.9 (4)
C7—P1—C1—C2	−108.8 (4)	C25—N1—C26—C27	−118.3 (5)
C13—P1—C1—C2	148.7 (3)	C33—N1—C26—C27	63.9 (5)
Pt1—P1—C1—C2	20.8 (4)	N1—C26—C27—C28	56.3 (6)
C6—C1—C2—C3	0.4 (7)	N1—C26—C27—C32	−125.6 (5)
P1—C1—C2—C3	−178.1 (4)	C32—C27—C28—C29	0.6 (8)
C1—C2—C3—C4	0.1 (8)	C26—C27—C28—C29	178.7 (4)
C2—C3—C4—C5	−0.5 (9)	C27—C28—C29—C30	−1.0 (8)
C3—C4—C5—C6	0.5 (10)	C28—C29—C30—C31	0.3 (10)
C4—C5—C6—C1	−0.1 (9)	C29—C30—C31—C32	0.7 (10)
C2—C1—C6—C5	−0.4 (7)	C28—C27—C32—C31	0.4 (8)
P1—C1—C6—C5	177.9 (4)	C26—C27—C32—C31	−177.8 (5)
C1—P1—C7—C8	−21.8 (4)	C30—C31—C32—C27	−1.0 (9)
C13—P1—C7—C8	86.5 (4)	C25—N1—C33—P2	−51.3 (5)
Pt1—P1—C7—C8	−147.4 (3)	C26—N1—C33—P2	126.5 (4)
C1—P1—C7—C12	166.9 (3)	C40—P2—C33—N1	112.4 (4)
C13—P1—C7—C12	−84.7 (4)	C34—P2—C33—N1	−148.1 (4)
Pt1—P1—C7—C12	41.4 (4)	Pt1—P2—C33—N1	−27.9 (4)
C12—C7—C8—C9	1.5 (7)	C40—P2—C34—C35	−114.5 (4)
P1—C7—C8—C9	−169.5 (4)	C33—P2—C34—C35	138.4 (4)
C7—C8—C9—C10	−0.9 (8)	Pt1—P2—C34—C35	16.6 (4)
C8—C9—C10—C11	0.4 (9)	C40—P2—C34—C39	61.3 (4)
C9—C10—C11—C12	−0.7 (9)	C33—P2—C34—C39	−45.8 (4)
C10—C11—C12—C7	1.4 (8)	Pt1—P2—C34—C39	−167.7 (3)
C8—C7—C12—C11	−1.8 (7)	C39—C34—C35—C36	−0.1 (8)
P1—C7—C12—C11	169.8 (4)	P2—C34—C35—C36	175.8 (4)
C21—N3—C13—P1	73.8 (5)	C34—C35—C36—C37	1.0 (9)
C14—N3—C13—P1	−107.6 (4)	C35—C36—C37—C38	−1.0 (9)
C7—P1—C13—N3	47.1 (3)	C36—C37—C38—C39	0.0 (9)
C1—P1—C13—N3	157.5 (3)	C37—C38—C39—C34	0.9 (8)
Pt1—P1—C13—N3	−78.9 (3)	C35—C34—C39—C38	−0.8 (7)
C21—N3—C14—C15	74.5 (5)	P2—C34—C39—C38	−176.8 (4)
C13—N3—C14—C15	−104.2 (4)	C34—P2—C40—C45	−121.8 (4)
N3—C14—C15—C20	32.0 (6)	C33—P2—C40—C45	−18.9 (4)
N3—C14—C15—C16	−151.7 (4)	Pt1—P2—C40—C45	116.2 (4)
C20—C15—C16—C17	0.6 (6)	C34—P2—C40—C41	60.7 (4)
C14—C15—C16—C17	−175.9 (5)	C33—P2—C40—C41	163.6 (3)
C15—C16—C17—C18	0.0 (8)	Pt1—P2—C40—C41	−61.2 (4)
C16—C17—C18—C19	−1.0 (9)	C45—C40—C41—C42	−0.5 (6)
C17—C18—C19—C20	1.3 (9)	P2—C40—C41—C42	177.2 (3)
C16—C15—C20—C19	−0.2 (6)	C40—C41—C42—C43	0.2 (7)
C14—C15—C20—C19	176.2 (4)	C41—C42—C43—C44	0.3 (9)
C18—C19—C20—C15	−0.7 (8)	C42—C43—C44—C45	−0.4 (9)
C25—N2—C21—N3	−173.3 (4)	C41—C40—C45—C44	0.3 (7)
C25—N2—C21—C22	5.2 (5)	P2—C40—C45—C44	−177.1 (4)
C13—N3—C21—N2	4.5 (6)	C43—C44—C45—C40	0.1 (8)

### Hydrogen-bond geometry (Å, °)

**Table:**

Cg1 is the centroid of C15–C20 benzene ring.

**Table:**

*D*—H···*A*	*D*—H	H···*A*	*D*···*A*	*D*—H···*A*
C5—H5···Cg1^i^	0.93	3.00	3.812 (2)	146
C26—H26B···Cg1^ii^	0.97	2.96	3.807 (2)	147

Symmetry codes: (i) −*x*+3/2, −*y*+1, *z*+1/2; (ii) −*x*+3/2, −*y*+1, *z*−1/2.
